# Top-Down Controls of Bacterial Metabolism: A Case Study from a Temperate Freshwater Lake Ecosystem

**DOI:** 10.3390/microorganisms10040715

**Published:** 2022-03-25

**Authors:** Angia Sriram Pradeep Ram, Marie-Eve Mauduit, Jonathan Colombet, Fanny Perriere, Antoine Thouvenot, Télesphore Sime-Ngando

**Affiliations:** 1 Laboratoire Microorganismes: Génome et Environnement, UMR CNRS 6023, Université Clermont-Auvergne, CEDEX, 63178 Aubière, France; jonathan.colombet@uca.fr (J.C.); fanny.perriere@uca.fr (F.P.); telesphore.sime-ngando@uca.fr (T.S.-N.); 2 Athos Environnement, 112 Avenue du Brézet, 63100 Clermont Ferrand, France; marie-eve.mauduit@athos-environnement.fr (M.-E.M.); antoine.thouvenot@athos-environnement.fr (A.T.)

**Keywords:** bacteria, viruses, lytic viral infection, viral production, heterotrophic nanoflagellates, bacterial growth efficiency, freshwater lake

## Abstract

In freshwater environments, limited data exist on the impact of mortality forces (viruses and heterotrophic nanoflagellates) on bacterial growth efficiency (BGE, index of bacterial carbon metabolism) compared to resource availability. An investigation to determine the relative influence of viral lysis and flagellate predation (top-down forces) on BGE was conducted in a mesotrophic freshwater system (Lake Goule, France) with time and space. Viral abundance was significantly (*p* < 0.001) related to bacterial abundance by a power law function with an exponent less than 1, emphasizing that the increases in host population (bacteria) together with viruses were not proportionate. A lytic viral strategy was evident throughout the study period, with high lysis of the bacterial population (up to 60%) supported by viral production rates. Viral processes (lysis and production) that were influenced by bacterial production and heterotrophic nanoflagellate abundance had a positive impact on BGE. Estimates of BGE were variable (9.9–45.5%) due to uncoupling between two metabolic parameters—namely bacterial production and respiration. The existence of a synergistic relationship between viruses and flagellates with bacteria in Lake Goule highlighted the decisive impact of top-down agents in sustaining the bacterial carbon metabolism of non-infected population through the nature of vital resources released via mortality processes.

## 1. Introduction

Freshwater systems are unique habitats in which the prevailing physical, chemical and biological environment enables broad and phylogenetically diverse microbial communities with high ecological plasticity to thrive [1]. Among these, viruses, which constitute numerous, ubiquitous and diverse microbial communities with high abundances of 10^6^–10^8^ viruses per ml in freshwaters, are the most infectious agents, capable of infecting unicellular life forms (mainly bacteria) that form the base of the aquatic food web [2,3]. As an actor of cellular lysis, viruses can have a pivotal impact on bacterial communities by influencing their evolutionary dynamics, bacterial-driven biogeochemical processes, energy transfer across trophic levels and greenhouse gas emissions [4,5,6]. Their potential ability to destroy about 40–60% of the total bacterial biomass on a daily basis in freshwaters has substantiated their role as an important mortality source or top-down control agent apart from protistan bacterivory [7]. The release of cellular material rich in organic carbon and inorganic nutrients in the surrounding water following successful viral-mediated bacteriolysis is principally processed by the non-infected populations in two major ways: by bacterial biomass production (i.e., bacterial production, BP) and by the remineralization of assimilated organic carbon to CO_2_ (bacterial respiration, BR), which is expressed as bacterial growth efficiency (BGE). BGE is a proxy of bacterial carbon metabolism that evaluates the fate of organic inputs (link or sink) in aquatic systems [8].

Apart from major abiotic factors such as temperature and resource availability (inorganic and organic nutrients), the impacts of trophic interaction such as viral lysis on BGE are less well studied. Viruses can have contrasting effects on community BGE, either negatively through the selective lysis of active cells or positively (viral shunt) through resource supply to support non-infected or viral-resistant bacterial populations. Few investigations linking viral infection to BGE have yielded two contrasting conclusions, with reports suggesting either a potential opposite impact on BGE through subsequent lysis of active cells [9,10] or no effects [11]. The viral impact on BGE is less studied than its effect on the bacterial community structure [12,13] and substrate supply [14,15]. Since freshwater lakes experience low nutrient concentrations at some point of the year, it could be hypothesized that the type of limiting nutrient could perhaps define the ecological importance of viruses as bottom-up or top-down agents in influencing bacterial carbon metabolism. Few quantitative data have been published in freshwater systems on the relative influence of phage infection and their lyses on bacterial carbon metabolism [16,17,18], thus leaving many questions unanswered.

Virus–bacteria interactions are highly complex, and freshwater-based investigations on the regulation of metabolic activity of bacterioplankton through its parameters—namely, production and respiration by viruses—remain sparse. Since the importance of viruses has been highlighted in many lake ecosystems, we hypothesize that they should play a crucial role (positive or negative effect) in carbon sequestration. Although larger emphasis has been placed on viral lysis in the control of bacterial metabolism, heterotrophic nanoflagellate grazing potential has also been taken into account as it also impacts bacterial communities through their prey morphology and feeding preferences [19]. In addition, the grazing of crustacean zooplankton such as *Daphnia* [20] and the possible loss of bacteria by predatory bacteria such as *Bdellovibrio* [21] can also impact the dynamics of bacterial populations in freshwater systems.

In aquatic systems, host specificity and the size-selective mortality of bacteria by viruses and flagellates, respectively, can impact the growth efficiency of non-targeted bacterial population. We consider that such a combined activity of top-down forces generates growth resources (especially limiting nutrients) for bacteria; as a consequence, synergistic interactions could be expected among grazer, bacterial and viral activities. The main objective of the study was to specifically determine the relative influence of viral lysis and flagellate predation on the carbon flux passing through bacteria, for which we collected data on bacterial metabolic parameters (production and respiration) together with viral processes (phage infection and viral production) in a Central France lake ecosystem. In this study, we evaluated the link between BGE and mortality forces and determined to what extent they exert their impact on carbon cycling in the chosen freshwater ecosystem. 

## 2. Material and Methods

### 2.1. Study Location and Sample Collection

Lake Goule, situated in Central France, was created in 1838 and originally intended to supply water to steel companies located downstream. The lake is fed by two low flow streams—namely, Auron and Evy. The hydrological functioning of Lake Goule is characterized by a strong variation in the water level during the summer. Characteristics of Lake Goule are detailed in Table 1.

Water samples were collected (in triplicates) from the designated point of the lake (46°73′ N, 2°79′ E) during the period between March and October (2018) at the euphotic (integrated samples) and aphotic (1 m above the sediments) zones using a hose pipe sampler and 10 L Van Dorn bottle, respectively. The collected samples were pre-filtered through a 150 µm nylon mesh to remove larger organisms and transported on ice and in the dark to a laboratory (within 2 h), where they were immediately processed for chemical, chlorophyll *a* and microbiological analysis upon arrival. Euphotic depth (Zeu), which was determined from Secchi measurements (Z_SD_), ranged between 1.1 m and 2.5 m during the study period, and the integrated samples collected in the euphotic zone could largely be representative of the surface layer. Euphotic depth was calculated using the relationship Zeu = 2.42 Z_SD_, with an assumption that the Secchi depth is the 15% light penetration depth [22]. 

### 2.2. Physicochemical Analysis

A multiparameter probe (WTW-OXI 320, Harfsen, The Netherlands) was used to measure profiles of in situ water temperature, pH, conductivity and dissolved oxygen along the water column. Ammonium and orthophosphate concentrations were determined spectrophotometrically by indophenol blue and molybdenum blue methods, respectively [23]. Nitrate and nitrite were analyzed photometrically by continuous flow analysis [24]. Total organic carbon (lakewater filtered through 150 µm mesh) and dissolved organic carbon (lakewater passed through pre-combusted Whatman GF/F filters) concentrations together with total dissolved nitrogen (TDN) were analyzed with a Shimadzu total organic carbon and nitrogen analyzer [25]. Dissolved organic nitrogen (DON) was determined by subtracting the inorganic nitrogen (nitrate, nitrite and ammonia) concentration from TDN. Samples for total chlorophyll *a* concentration (Chl *a*) were obtained after filtration of 400–500 mL lake water through Whatman GF/F filters. Chlorophyll pigments were extracted in 90% acetone for 24 h in the dark at 4 °C, and their concentrations were measured by the spectrophotometric method [26].

### 2.3. Microbial Abundances

For flow cytometric determination of viral and bacterial abundances, 1 mL aliquots of samples were fixed with paraformaldehyde (0.5% final concentration) for 30 min in the dark at 4 °C. Samples were run instantly on a FACS Aria Fusion SORP flow cytometer (BD Sciences, San Jose, CA, USA) equipped with an air-cooled laser providing 50 mW at 488 nm with a 502 long pass and 530/30 band pass filter set-up. Samples were diluted using 0.02 µm filtered TE buffer (10 mM Tris–HCl and 1 mM EDTA, pH 8). For viral counts, samples were stained in the dark for 5 min with SYBR Green I (Molecular Probes, Hillsboro, OH, USA) at a final ×10^−4^ dilution of commercial stock and heated at 80 °C in the dark for 10 min. The samples were allowed to cool for 5 min prior to analysis. Viral populations were enumerated and identified based on right-angle scatter (SSC) and green fluorescence (530 nm) based on an established protocol [27]. For bacterial counts, samples were stained in the dark for 15 min using SYBR Green I at a final concentration of 1 × 10^−4^ before analysis [28]. 

In the cytogram, the two major bacterial sub-populations, referred to as high (HNA) and low nucleic acid (LNA) communities, were classified based on the differences in the side scatter plot and relative fluorescence [29]. The list modes were analyzed using CellQuest Pro software package (BD Biosciences, San Jose, CA, USA; version 4.0).

Heterotrophic nanoflagellates (HNFs) were enumerated by epifluorescence microscopy in glutaraldehyde fixed (1% final concentration) water samples. Briefly, 20 mL of samples stained with DAPI (4,6-diamidino 2-phenylindole) at a final concentration of 10 µg mL^−1^ and dark-incubated for 20 min were filtered onto 0.8 µm polycarbonate black filters (25 mm) [30]. A minimum of 50 fields with 75–100 HNFs were counted using an epifluorescence microscope (LEICA DC 300 F, Leica Microsystems, Wetzlar, Germany) at ×1000 magnification equipped with optical filters for UV. HNFs were identified by their size and presence of flagella. An average flagellate clearance rate of 2.1 nL ind^−1^ h^−1^ deduced from our previous study was used to calculate flagellate grazing potential [7].

### 2.4. Bacterial Metabolic Parameters 

Bacterial respiration (BR) was measured from oxygen uptake by Winkler’s method in lake water samples filtered through 1.0 µm polycarbonate (47 mm) filters (Whatman, Little Chalfont, UK). The filtrate was carefully transferred to calibrated borosilicate glass bottles (150-mL capacity) without introducing air bubbles. Time zero (initial) and 24 h (final) incubated (at in situ temperature conditions) bottles (replicates) were fixed with Winkler’s reagents for measurement of dissolved oxygen (DO) concentrations based on endpoint detection. BR was calculated by subtracting DO concentration between initial and final replicate bottles. The oxygen values were multiplied by 0.375 to convert into carbon equivalents with a RQ of 1 [31].

Bacterial production (BP) was deduced from growth rate (µ), which was determined by the dilution technique [32]. The water samples filtered through a 1.0 µm polycarbonate filter (Whatman, Little Chalfont, UK) were diluted with four parts of 0.02 µm filtered lake water and dark-incubated for a 24 h period at in situ temperature conditions. µ (d^−1^) was calculated based on the number of cells that produced ΔN during incubation time t, and cell concentration at the beginning N_0_ as

µ = t^−1^ × ln(1 + ΔN × N_0_^−1^) (1)

BP (cells L^−1^ d^−1^) was converted to carbon equivalents by using a mean carbon value of 20 fg C per cell [18].

To avoid contamination of lake water samples, all necessary precautions were taken, such as using acid-washed tubing, glass bottles and filtration equipment, evaluating the efficiency of chosen pore size filters, frequent filter replacement to avoid clogging, filtration under low light condition and using a low filtration pressure (<50 mm Hg) to avoid cell disruption.

We calculated bacterial growth efficiency (BGE) as [BP/(BP + BR)] × 100.

### 2.5. Frequency of Visibly Infected Bacterial Cells (FVIC) and Viral Morphotypes 

Phage infection required to determine viral-mediated bacterial mortality was estimated by the FVIC approach using transmission electron microscope (TEM) analysis [33]. Bacterial cells in formalin-fixed samples (final conc. 2% *v*/*v*) were directly deposited onto TEM grids (Formvar carbon-coated 400-mesh) by ultracentrifugation using a Swing-Out-Rotor (Optima LE-80K, Beckman Coulter SW40 Ti) for 20 min at 70,000× *g*. The grids were individually stained with 2% (*w*/*v*) uranyl acetate for 30 s, after which they were rinsed with ultrapure water and immediately wicked away with absorbent paper. Grids were examined with TEM (JEM-2100 Plus TEM, Tokyo, Japan) at 8000–40,000× magnification with an accelerating voltage of 80 kV. A minimum of 400 bacterial cells were counted per grid to determine FVIC. A bacterial cell was scored as infected when at least three or more phages were visualized inside the cell. FVIC counts were converted to the frequency of infected cells (FICs) and viral-induced bacterial mortality (VIBM) using the empirical equations of Weinbauer [34] (FIC = 9.524 × FVIC − 3.256) and Binder [35] (VIBM = (FIC + 0.6 × FIC^2^)/1 − 1.2 × FIC), which relates to the bacteriophage infection cycle. Burst size was estimated by enumerating the viral particles in infected bacterial cells using TEM. 

Phages were identified and grouped as myoviruses, podoviruses, siphoviruses and non-tailed viruses based on their size, capsid morphology and tail characteristics (if present) using a JEM-2100 Plus TEM (Tokyo, Japan) at a magnification of 50,000–80,000×.

### 2.6. Viral Production (VP)

VP was assessed by the modified dilution approach [36]. Fifty milliliters of water samples (in triplicates) was mixed with 100 mL of 0.02 µm filtrate (virus free) in glass bottles and dark incubated. Subsamples were collected at regular time (t in hours) intervals: i.e., t0, t2, t4, t6 and t8. Subsamples were fixed immediately with 0.5% paraformaldehyde and consequently enumerated for viral abundance by flow cytometry (see previous section). VP was calculated from a linear regression between VA versus time and expressed in hours.

### 2.7. Data Analyses

Statistical analyses were executed using Minitab Version 17 (Minitab Inc., State College, PA, USA). Comparison of multiple mean values was conducted by using an analysis of variance (ANOVA). Potential product moment analysis was used to examine the simple correlation among the measured environmental and microbiological parameters. The significance of performed statistical analyses was accepted at an alpha value of *p* < 0.05. 

## 3. Results

### 3.1. Physico-Chemical Environment

Means and ranges of abiotic parameters of the sampled euphotic and aphotic zones during the study period are listed in Table 2. Water temperature measured along the water column varied seasonally, with values increasing from 8.6 (March) to 28.2 °C (August) and then gradually decreasing to 17.5 °C in October in the euphotic zone. This pattern was also reflected in the aphotic depths, but with decreased values (Supplementary Materials Figure S1). Thermal stratification occurred between June and August, where the difference in water temperature between euphotic and aphotic depths was >4 °C. The euphotic zone was characterized by oxic conditions (>6.8 mg O_2_ L^−1^) throughout the study period, whereas anoxia was detected between June and August at the aphotic depths (Mean ± SD = 0.57 ± 0.12 mg O_2_ L^−1^) (Supplementary Materials Figure S1). Among the inorganic nutrients, nitrate and phosphate concentrations were less than 14.3 µmol L^−1^ and 0.3 µmol L^−1^ during the summer months (June–August), comparable to limited conditions in lake ecosystems. Overall total (TOC) and dissolved organic carbon (DOC) concentrations did not vary with seasons and depths, with a coefficient of variation (CV) less than 15%. DOC contributed to 68% to 93% of TOC (Mean ± SD = 1033.3 ± 133.3 µmol L^−1^), suggesting variable rates of organic material decomposition, which could potentially be bioavailable for microbial processing. DOC:DON and atomic N:P ratios showed a mean value of 15.7 ± 2.9 and 48.2 ± 32.9, respectively, irrespective of depth and seasons. Overall chlorophyll *a* concentration ranged between 0.1 (May) and 23.1 µg L^−1^ (August), with no significant differences between the studied depths (Table 2).

### 3.2. Standing Stocks

**Figure 1:**
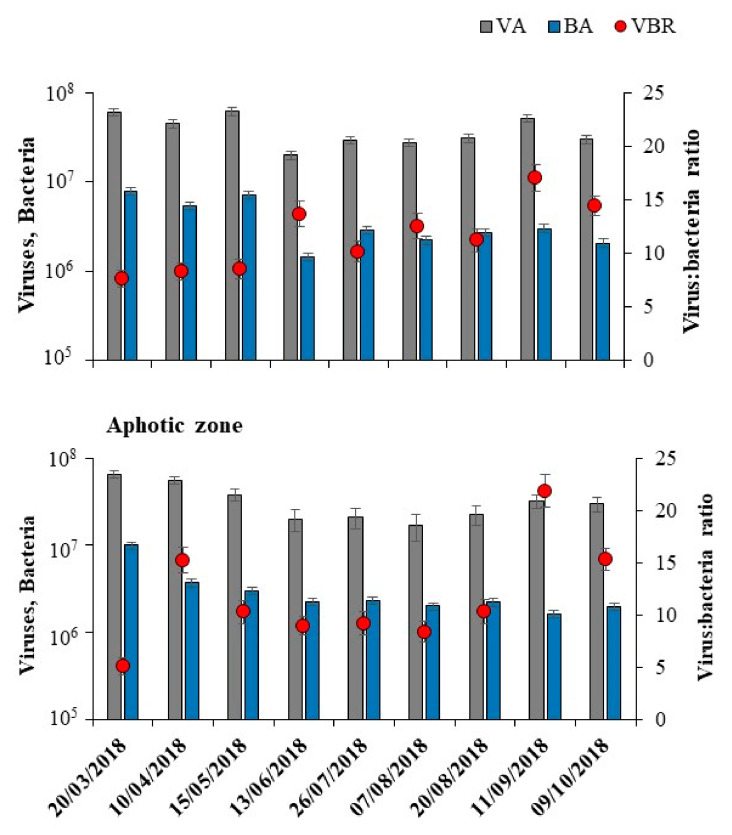
Time series variations in the abundance of viruses (VA; grey bars), bacteria (BA; blue bars) and their ratio (VBR; red dots; right *y* axis) at sampled depths in Lake Goule. Data represent mean ± SE (*n* = 3).

Microbial standing stocks and their mediated activity in Lake Goule are presented in Table 3. Across the studied months and depths, viral abundance (VA) was on average 10-fold higher than bacterial abundance (BA), but temporal changes in VA and BA were similar (Figure 1). For VA, the highest value that was observed in the early spring (March) at euphotic (6.0 × 10^7^ mL^−1^) and aphotic (6.6 × 10^7^ mL^−1^) zones coincided with the peaks in BA (Figure 1), when nitrate concentrations were at the highest (1.51 mg L^−1^). In the euphotic zone, the maxima of VA and BA in March were about four and five-fold higher, respectively, than the lowest abundances obtained in June (VA = 2.0 × 10^7^ mL^−1^; BA = 1.5 × 10^6^ cells mL^−1^) (Figure 1). Virus to bacteria ratio (VBR), which is a proxy of viral activity, showed a mean of 11.5 and 11.6 at euphotic and aphotic zones. The variation in VBR (5.1–21.9) was mainly due to high fluctuations in BA (CV = 79%) relative to VA (CV = 45%). Overall, VA was better described as a power law function of BA with fitted scaling exponents of less than 1 (VA = 16.54 BA^0.65^, r^2^ = 0.75, *p* < 0.001, n = 18), indicating that BA was a strong predictor of VA, accounting for 75% of the variation (Supplementary Materials Figure S2). Both VA and BA were correlated to dissolved nitrogen rather than to organic carbon and chlorophyll concentrations (Table 4). Among the bacterial physiological groups, low nucleic bacteria (LNA) were dominant (mean = 3.0 × 10^6^ cells mL^−1^) throughout the study period, comprising about 77% of the total bacterial abundance compared to the high nucleic acid bacterial population (HNA, mean = 0.67 × 10^6^ cells mL^−1^). The LNA subgroup dominated during the period of inorganic phosphate limitation, which was reflected in a high N:P ratio (>40).

HNFs were three orders of magnitude lower than BA, with higher (*p* < 0.01) abundance in the euphotic (1.8 ± 0.7 × 10^3^ mL^−1^) than in the aphotic (1.0 ± 0.4 × 10^3^ mL^−1^) depths (Table 3). Temporal changes in HNFs were small, and the fluctuations were not similar to patterns in VA and BA.

### 3.3. BP, BR and BGE

**Figure 2:**
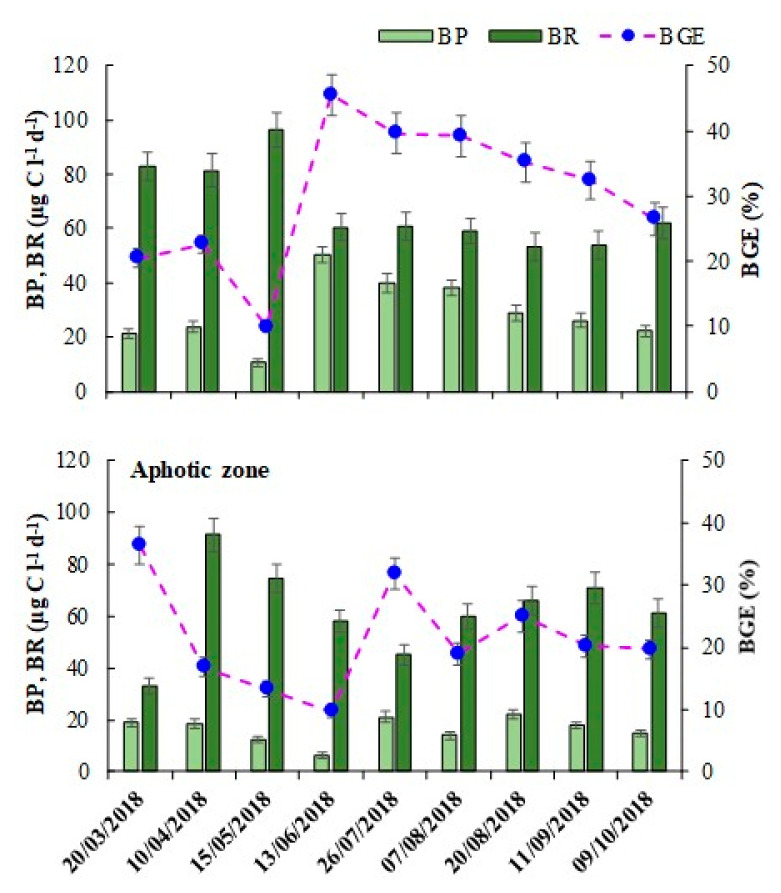
Time series variations in bacterial production (BP; light green bars), bacterial respiration (BR; dark green bars) and estimates of bacterial growth efficiency (BGE; blue dots; right *y* axis) at euphotic and aphotic zones in Lake Goule. Data represent mean ± SE (*n* = 3).

Overall BP varied between 6.2 and 50.5 µg C L^−1^ d^−1^, with significantly higher (*p* < 0.001) values in the euphotic (29.1 ± 11.9 µg C L^−1^ d^−1^) compared to aphotic zone (16.7 ± 5.0 µg C L^−1^ d^−1^). In the euphotic zone, high BP was observed in the summer months (June–August) (Figure 2), whereas such seasonal differences were not evident in the aphotic zone. BR ranged between 33.3 and 96.3 µg C L^−1^ d^−1^, and unlike BP, the values did not vary significantly with depths and seasons (Table 3). BR was apparently higher (*p* < 0.001) than BP by 2.3 and 3.6-fold at euphotic and aphotic zones, respectively. BGE, calculated from BP and BR estimates, ranged from 9.9 to 45.6, with significantly (*p* < 0.05) higher values in the euphotic (30.2 ± 11.2%) compared to aphotic zone (22.3 ± 8.5%). The observed variation in BGE at sampled layers was due to the variability in BP (CV = 49%) rather than that of BR (CV = 24%). Uncoupling between BP and BR explained the observed variability in BGE; as a result, BGE could easily be predicted from BP values (y = 0.86x + 6.26, r^2^ = 0.79, *p* < 0.001, *n* = 18, Supplementary Materials Figure S3) alone. BP and BGE were strongly correlated (*p* < 0.001) to DOC concentration, chlorophyll concentration and HNF abundance (Table 4). Regression analysis suggested that no significant relation was evident between BP and BA at the studied depths. 

### 3.4. Lytic Viral Infection and Production Rates

**Figure 3:**
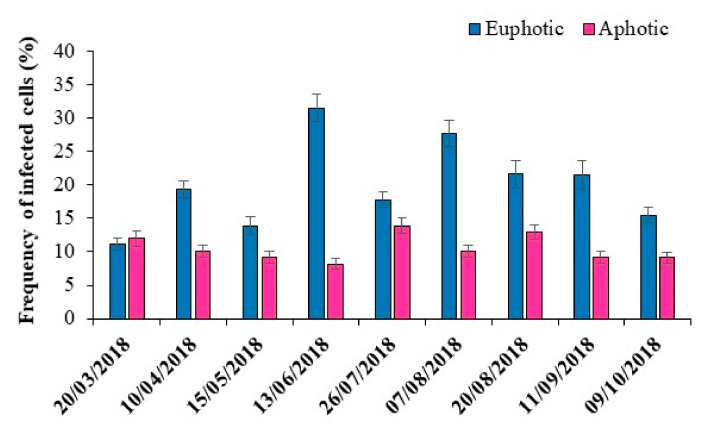
Time series variations in frequency of viral-infected bacterial cells (FIC) at euphotic and aphotic zones in Lake Goule. Data represent mean ± SE (*n* = 3).

Viral lytic infection by the FVIC approach using TEM was detected for all sampled occasions. Lytic infection was 2.8 and 1.7-fold higher than the lowest values obtained at the euphotic and aphotic depths, respectively (Figure 3). The calculated frequency of viral infected bacterial cells (FIC) varied from 8.2% to 31.5%, with a significantly higher percentage of infection (*p* < 0.001) in the euphotic (20.0 ± 6.5%) compared to aphotic zone (10.5 ± 2.0%). High infection rates that occurred in June (31.5%) coincided with the highest abundance of HNF (3.2 × 10^3^ cells ml^−1^) rather than the maxima of BA. The absence of a significant correlation or pattern between VA and FIC in Goule indicates that viral attack could be rather dependent on the density of the susceptible bacterial community than the whole host population density. FIC was positively correlated with BGE (*p* < 0.001, Supplementary Materials Figure S4) along with abiotic parameters such as temperature (*p* < 0.0.05) and DOC (*p* < 0.05) (Table 4). Multiple regression analysis indicated that BP and HNF were significant (*p* < 0.05) predictor variables that explained 79% of the variation in FIC (FIC = 2.3 + 0.45 × BP + 1.91 × HNF, r^2^ = 0.79, *p* < 0.001, *n* = 18). 

The average burst size estimates in the euphotic and aphotic zones were 22.8 and 14.6 bacterium^−1^, respectively. Myoviruses was the most dominant viral morphotype, followed by podo, sipho and untailed viruses (data not shown). The capsid diameter of viruses ranged between 35 and 120 nm, with a mean head size of 55 nm, suggesting that they were mainly bacteriophages. 

Viral production (VP) showed a similar trend to the observed percentage of lytic infection, with higher production rates (*p* < 0.001) in the euphotic (17.4 ± 4.5 × 10^5^ mL^−1^ h^−1^) than in aphotic (3.7 ± 1.1 × 10^5^ mL^−1^ h^−1^) zone (Table 3). VP was correlated to FIC (*p* < 0.001), BP (*p* < 0.001) and HNF (*p* < 0.01) but to none of the abiotic parameters (Table 4). 

### 3.5. Bacterial Mortality

Overall, viruses dominated heterotrophic nanoflagellates as a source of bacterial mortality on many occasions (June–October). The calculated viral lysis ranged between 9.5% and 60%, whereas the flagellate grazing or bacterivory potential, which varied from 0.5 to 2.9 × 10^5^ bacteria mL^−1^ d^−1^, accounted for between 6% and 82% of bacterial mortality at the studied depths (Table 3). Viral lysis exceeded grazer mortality by 2.5 and 1.2-fold in euphotic and aphotic zones, respectively. Both flagellate grazing potential and viral-induced bacterial mortality were positively related (*p* < 0.001), asserting the importance of viral lysis and flagellate predation on the bacterial community in Lake Goule.

## 4. Discussion

The present investigation, which was carried out in a freshwater system, is one of the few aiming to bring out the potential role of top-down forces in regulating bacterial metabolism (referred to as BGE). Our remarkable finding in the case of Lake Goule was that top-down regulation on BGE was evident based on the regression of bacterial abundance and production [37,38], which perhaps might be linked to resource (substrate supply) generation by top-down forces that essentially stimulate the growth and activity of non-targeted or viral resistant populations (defense specialists).

### 4.1. Standing Stock and Lytic Infection

Flow cytometry signatures of viral and bacterial abundances revealed that their counts were within the reported range of values for mesotrophic freshwater systems [39,40,41], with no marked fluctuation in their ratio with time and space. Irrespective of the sampled depths, viral abundance was better described by a power law function of bacterial abundance with a scaling exponent less than 1, unlike reports from other freshwater systems [7,42] indicating that viral abundance increases less than proportionately given increases in bacterial abundances as previously reported for nutrient limited systems [43]. Viral lysis results in the release of nutrients to the surrounding environments with a higher N:P ratio compared to bacteria [44]; as a consequence, the impact of phages on bacterial communities would depend on nutrient concentration. The emergence of a significant relationship between viruses and bacteria that was better explained by a power-law coefficient are consistent with analyzed data from global oceanic systems [45]. The observed negative correlation between the virus to bacteria ratio and bacteria (Table 3), which is considered to be a hallmark of the power-law relationship, has been attributed to episodic events such as virus-induced termination of phytoplankton blooms or induction of lysogenic populations that influence total bacterial and viral counts [45]. We principally focused on lytic viruses, which through the rapid production of viral particles directly contribute to viral shunt rather than dormant temperate viruses. Viral lytic infection determined by the FVIC approach through direct observation using TEM in Lake Goule (1.2 to 3.7%) was comparable and within the typical range of those (i.e., <5%) reported for freshwater environments [7,41,46]. In contrast to reports from other freshwater systems, the absence of a relationship between the percentage of viral-infected cells and both viral and bacterial abundances indicates that the viral attack was dependent on the density of the susceptible community, rather than the density of the total host population. The incongruity between lytic infection and host density, which has been referred to as the infection paradox, is known to occur if the microbial community is more diverse or if a proportion of each host population is resistant to a co-occurring virus. The high mortality of up to 60% of the bacterial community in Lake Goule indicated that cell biomass was intensively redirected back to the pool of potential substrates via the viral shunt. Lytic infection and viral production, which were determined through independent approaches, were robustly correlated (r = 0.86, *p* < 0.001, Supplementary Materials Figure S4) with each other, suggesting that these viral processes should have an influential impact on carbon sequestration by bacteria. 

### 4.2. Top-Down Impact on Bacterial Growth Efficiency

BGE is a physiologically relevant trait that refers to the metabolism of the bacterial community and offers biogeochemical information on the flux of carbon passing through bacteria up to a higher trophic level. The observed variability in BGE (9.9% to 45.5%) over the study period in Lake Goule was duly explained by the uncoupling between bacterial production and respiration that provides metabolic flexibility to natural bacterioplankton in confronting fluctuating nutrient regimes especially observed in freshwater systems [8,47]. The variation in BGE coupled with the increase in bacterial production may have been essentially driven by the quality of the available DOM. The prevailing low substrate DOC:DON ratio (mean = 15.6 ± 3.0) indicates that heterotrophic bacteria could assimilate more nitrogen for their conversion into microbial protein, which is essential for their proliferation [48]. 

The perception that viruses are strongly linked to their major host (bacteria) in the Lake Goule was supported by a robust relationship (*p* < 0.001) between bacterial production and percentage of lytic infection, implying that the in situ production of viruses relies on the active group of the host community that contributes to high rates of bacterial production. Our findings agree with previous reports that lytic infection is primarily supported by increased host activity [49,50]. 

In addition to bacterial production, our statistical analysis indicated that HNFs and their potential grazing estimates were also a significant predictor of viral infection in Lake Goule. The contribution of bacterial production and HNFs as significant predictor variables (79%) of FIC in Lake Goule (Supplementary Materials Figure S4) are in agreement with the previous reports from Rimov Reservoir (Czech Republic) and Lake Biwa (Japan), where viral infection tended to be high when HNFs are high [51,52]. The release of labile nutrients through nutrient regeneration by the combined activity of top-down agents to support their host abundance and activity especially under nutrient limiting conditions could explain the scenario of increasing phage infectivity with increase in flagellate grazing pressure. The positive correlation between viral lytic infection and BGE that we found at least in Lake Goule might closely be linked to substrate supply, where viral shunt can release competition pressure among bacterial communities. Our studies find support from experimental studies using marine waters, where a high BGE was observed in microcosms supplemented with DOM originating from a bacterial compared to protistan community [53]. Our findings of a positive impact of viruses on BGE contrast with the reports from other freshwater systems [16,17]. Studies conducted in freshwater and marine systems have reported viral infection to decrease community BGE, which was largely explained by enhanced respiration rates or the energetic cost required to process regenerated DOM by non-targeted bacterial populations [9,18,54]. However, the above scenario was not evident in the studied freshwater system, where no relation was evident between viral infection and bacterial respiration. Among the total bacterial communities, the low nucleic acid bacterial subgroup, which is generally known to thrive in low nutrient environments, was subjected to enhanced viral lysis in the studied freshwater ecosystem. The potential role of this subgroup, which comprised about 70–80% of the total bacteria in Lake Goule, contributed to substantial heterotrophic activity, thereby suggesting the ecological importance of this physiological group in freshwater environments [55]. Selective lysis of susceptible populations could benefit the minor high nucleic acid bacterial subgroup, which is capable of exploiting pulses of regenerated nutrients more quickly and efficiently [56]. 

The growth efficiency of bacterioplankton has been strongly linked to the concentration and availability of organic matter. Viral shunt produces elements rich in C and, together with intense protistan bacterivory that releases labile N and P, the DOM produced could be of differing nutritional quality than that produced by phytoplankton. Strong bacterioplankton growth could be enhanced in microenvironments through top-down activity, which results in the release of altered DOM or regenerated nutrients [57]. Viral lysis products are known to contain dissolved DNA, a pool of labile dissolved free and combined D amino acids that can be efficiently incorporated into the bacterial biomass of a non-targeted community at increased efficiency to meet its nutritional demand [58]. Across aquatic systems, the impact of top-down effects on community BGE mediated by DOM alteration has been sparsely studied.

Although we have not characterized bacterial community composition, our previous published studies have asserted that certain bacterial populations belonging to taxa such as *Limnohabitans*, *Paucibacter* and *Pseudomonas* were more substrate-responsive in efficiently utilizing regenerated nutrients [59]. Bacteriodetes (r strategists) are 10 times more efficient in processing organic carbon and are known to contribute to higher BGE than other phyla [53]. From the above point of view, the characterization of a bacterial community (composition and diversity) is crucial since different bacterial groups that comprise varying physiological states can adopt different strategies to process DOM at varying level of growth efficiencies. 

The domination of viral lysis over potential bacterivory by HNF at most of the time provides strong evidence that viruses are a critical component in Lake Goule. Our results show that lytic dynamics alone can be consistent with a sublinear relationship between viruses and bacteria. The influence of viruses on the ecosystem could vary depending on the limiting nutrient. Although it was evident that viruses had a decisive impact on BGE, perhaps through substrate supply, insufficient information exists on the effects of resource generation in maintaining BGE. Experimental verifications (laboratory microcosm experiments) together with changes in bacterial community structure during the periods of nutrient repletion and depletion are required to determine the virus–bacteria interactions in such freshwater systems.

## Figures and Tables

**Table 1:** Hydrological and morphological characteristic of Lake Goule (France).

Sampling location	46°73′ N, 2°79′ E
Elevation (m)	220
Origin	Man made
Trophic status	Mesotrophic
pH	Alkaline (8.5)
Maximum depth (m)	9
Water circulation	Holomictic
Surface area (ha)	123
Storage volume (10^6^ m^3^)	3.8
Watershed area (km^2^)	35
Catchment: lake area ratio	28.5

**Table 2:** Physico-chemical characteristics of Lake Goule at the studied depths.

Parameters	Mean (Range) ^a^		*p* Value ^b^
	Euphotic	Aphotic	
Water temperature (°C)	20.0 (8.6–28.2)	18.1 (8.6–24.0)	NS
Dissolved oxygen (mg O_2_ L^−1^)	9.6 (6.8–13.1)	5.2 (0.5–12.1)	0.02
pH	8.5 (7.8–9.2)	8.2 (7.7–8.9)	NS
NH_4_-N (µmol L^−1^)	7.1 (3.6–21.4)	14.3 (3.6–35.7)	NS
NO_3_-N (µmol L^−1^)	28.6 (14.2–100.0)	28.6 (14.3–114.3)	NS
PO_4_-P (µmol L^−1^)	0.7 (und–1.3)	0.7 (und–1.3)	NS
Total organic carbon (µmol L^−1^)	1041.6 (816.7–1216.7)	1025.0 (808.3–1141.7)	NS
Dissolved organic carbon (µmol L^−1^)	841.7 (775.0–916.7)	825.0 (716.7–914.7)	NS
Total dissolved nitrogen (µmol L^−1^)	121.4 (92.9–185.7)	128.6 (100.0–207.1)	NS
DOC:DON ratio	14.7 (11.1–17.8)	16.7 (11.9–20.6)	NS
N:P ratio	50.5 (19.6–113.5)	45.9 (15.7–110.9)	NS
Chlorophyll *a* (µg L^−1^)	12.0 (0.1–23.1)	10.4 (0.2–18.8)	NS

^a^ *n* = 9 for each sampled layer. ^b^ Significance level of difference between euphotic and aphotic zone. und: under detection limits. NS denotes *p* values not significant.

**Table 3:** Microbial characteristics of Lake Goule at the studied depths.

Parameters	Mean (Range) ^a^		*p* Value ^b^
	Euphotic	Aphotic	
Viral abundance (10^7^ mL^−1^)	4.0 (2.0–6.2)	3.4 (1.7–6.6)	NS
Bacterial abundance (10^6^ cells mL^−1^)	3.9 (1.5–7.8)	3.2 (1.6–10.0)	NS
Virus to bacteria ratio	11.5 (7.7–17.0)	11.6 (5.1–21.9)	NS
Low nucleic acid bacteria (10^6^ cells mL^−1^)	3.1 (1.2–6.6)	3.0 (1.3–11.3)	NS
High nucleic acid bacteria (10^6^ cells mL^−1^)	0.7 (0.3–2.3)	0.6 (0.3–1.8)	NS
Bacterial production (µg C L^−1^ d^−1^)	29.1 (10.6–50.5)	16.7 (6.2–22.0)	0.009
Bacterial respiration (µg C L^−1^ d^−1^)	67.8 (53.4–96.3)	60.7 (33.3–91.4)	NS
Bacterial growth efficiency (%)	30.2 (9.9–45.5)	22.3 (9.7–36.3)	0.05
Frequency of infected cells (%)	20.0 (11.1–31.5)	10.5 (8.2–13.9)	0.001
Viral production (10^5^ mL^−1^ h^−1^)	17.4 (4.5–37.4)	3.7 (1.1–9.0)	0.001
Burst size (virus cell^−1^)	22.8 (5–71)	14.6 (5–32)	0.02
Heterotrophic nanoflagellate abundance (10^3^ cells mL^−1^)	1.8 (1.1–3.2)	1.1 (0.7–2.1)	0.01
Flagellate grazing potential (10^5^ cells mL^−1^ d^−1^)	2.9 (1.0–5.1)	1.9 (0.5–8.0)	NS
Viral induced bacterial mortality (%)	31.1 (13.7–60.2)	13.1 (9.5–18.1)	0.001
Bacterivory potential (%)	26.1 (8.4–75.2)	22.0 (5.8–81.9)	NS
Viral induced bacterial mortality/grazing potential	2.5 (0.2–7.1)	1.2 (0.2–2.9)	0.001

^a^ *n* = 9 for each sampled layer. ^b^ Significance level of difference between euphotic and aphotic zone. NS denotes *p* values not significant.

**Table 4:** Pearson correlation coefficient values among different environmental variables (*n* = 18).

	Temp	DOC	TN	Chl	BA	VA	HNF	BP	BR	BGE	FIC
DOC											
TN	−0.81 ***										
Chl											
BA	−0.72 ***		0.87 ***								
VA	−0.74 ***		0.77 ***	−0.45 *	0.87 ***						
HNF											
BP	0.53 *	0.60 **		0.50 *			0.72 ***				
BR				−0.64 **		0.59 **					
BGE	0.49 *	0.56 **		0.59 **			0.50 *	0.89 ***	−0.60 **		
FIC	0.51 *	0.53 *					0.72 ***	0.88 ***		0.78 ***	
VP							0.69 **	0.75 ***		0.59 **	0.87 ***

Temp: water temperature, DOC: dissolved organic carbon, TN: total nitrogen, Chl: chlorophyll concentration, BA: bacterial abundance, VA: viral abundance, HNF: heterotrophic nanoflagellate abundance, BP: bacterial production, BR: bacterial respiration, BGE: bacterial growth efficiency, FIC: frequency of viral infected bacterial cells, VP: viral production. Note: * *p* < 0.05, ** *p* < 0.01, *** *p* < 0.001.

## Supplementary Materials

The following supporting information can be downloaded at: https://www.mdpi.com/article/10.3390/microorganisms10040715/s1, Figure S1: Time series variations in water temperature (Temp) an dissolved oxygen (DO) concentration at euphotic and aphotic depths in Lake Goule; Figure S2: Relationship between bacterial and viral abundance in Lake Goule; Figure S3: Relationship of bacterial production with bacterial growth efficiency (A) and respiration (B) in Lake Goule; Figure S4: Relationship of viral infection with bacterial production (A) heterotrophic nanoflagellate abundance (B) and viral production (C) in Lake Goule.
