# Supplementary material for: Top-Down Controls of Bacterial Metabolism: A Case Study from a Temperate Freshwater Lake Ecosystem
Source: Microorganisms. 2022 Mar 25;10(4):715. doi: 10.3390/microorganisms10040715 (PMC9031129; doi:10.3390/microorganisms10040715)

Supplementary Materials Figure S1. Time series variations in water temperature (Temp) and dissolved oxygen (DO) concentration at euphotic and aphotic depths in Lake Goule

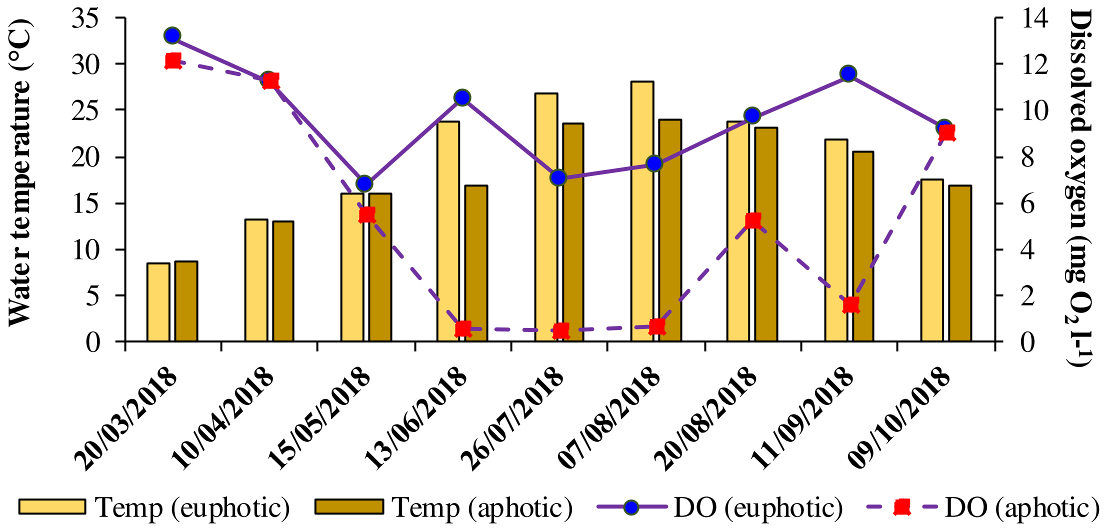

Supplementary Materials Figure S2. Relationship between bacterial and viral abundance in Lake Goule

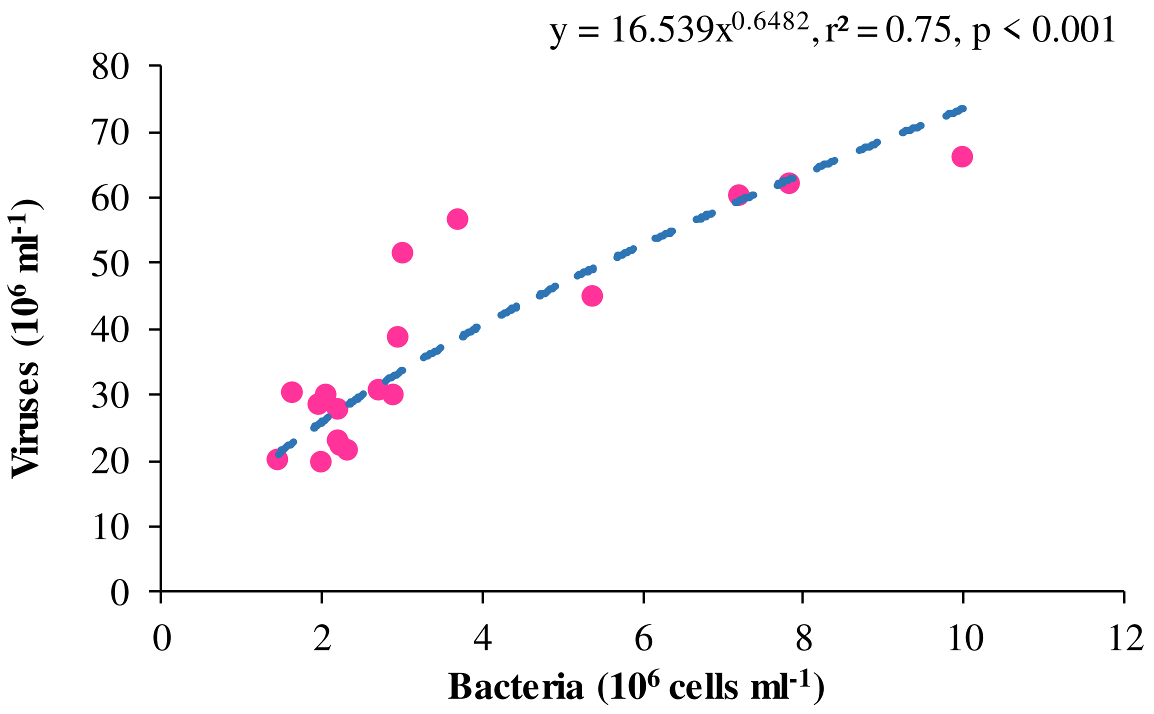

Supplementary Materials Figure S3. Relationship of bacterial production with bacterial growth efficiency (A) and respiration (B) in Lake Goule.

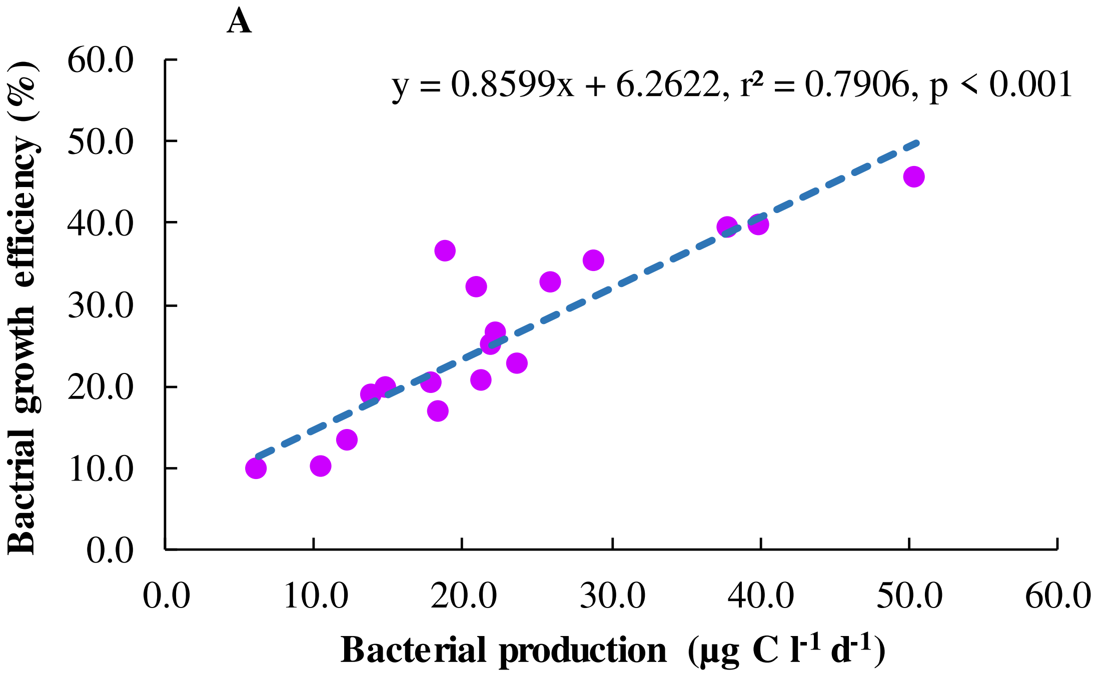

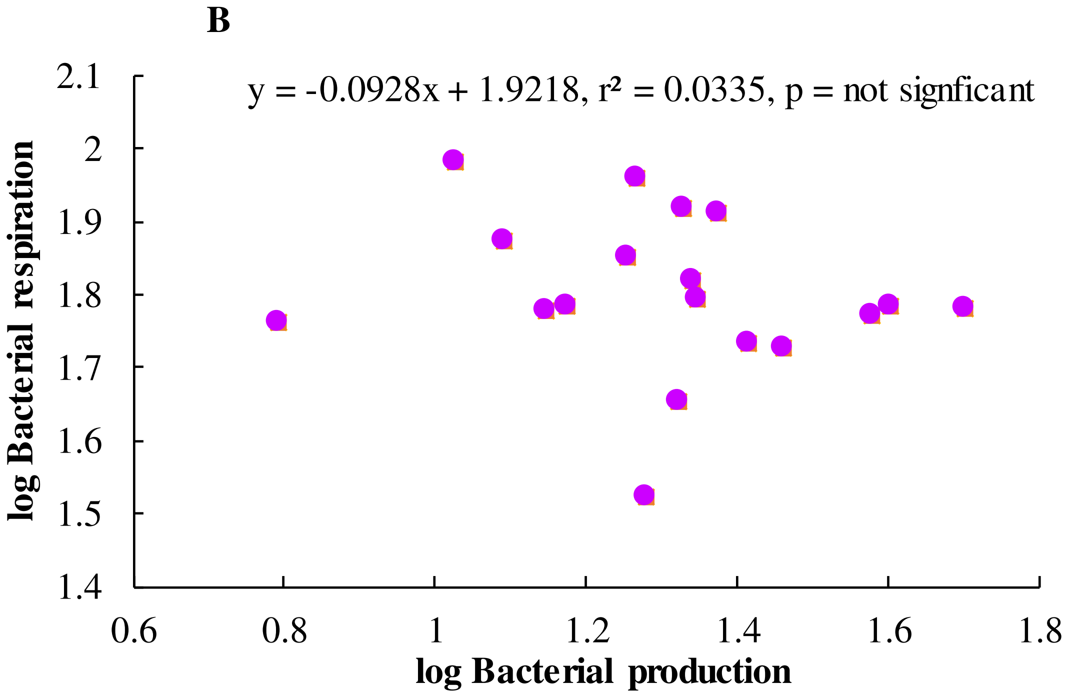

Supplementary Materials Figure S4. Relationship of viral infection with bacterial production (A) heterotrophic nanoflagellate abundance (B) and viral production (C) in Lake Goule.

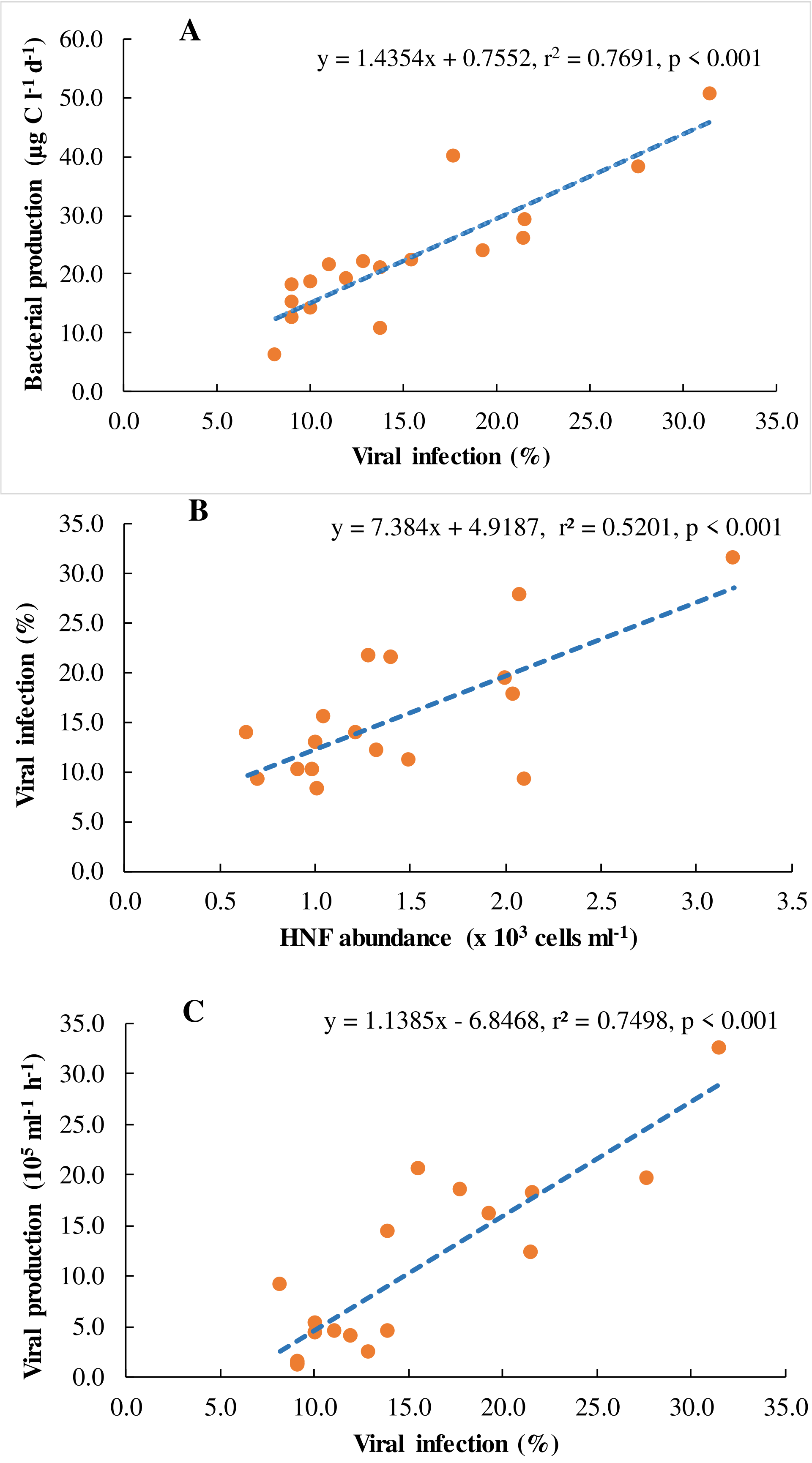

Supplement: Supplementary file 1 [file microorganisms-10-00715-s001.zip › microorganisms-1612702-supplementary.pdf]
